# Diagnosing Biliary Strictures: Distinguishing IgG4-Related Sclerosing Cholangitis From Cholangiocarcinoma and Primary Sclerosing Cholangitis

**DOI:** 10.1016/j.mayocpiqo.2021.03.005

**Published:** 2021-06-10

**Authors:** Yasuki Hori, Suresh T. Chari, Yoshihisa Tsuji, Naoki Takahashi, Dai Inoue, Phil A. Hart, Takeshi Uehara, Masayasu Horibe, Satoshi Yamamoto, Akira Satou, Lizhi Zhang, Kenji Notohara, Itaru Naitoh, Takahiro Nakazawa

**Affiliations:** a Department of Gastroenterology and Metabolism, Nagoya City University Graduate School of Medical Sciences, Nagoya, Japan; b Division of Gastroenterology and Hepatology, Mayo Clinic, Rochester, MN; c Department of Radiology, Mayo Clinic, Rochester, MN; d Department of Laboratory Medicine and Pathology, Mayo Clinic, Rochester, MN; e Department of General Medicine, Sapporo Medical University, Sapporo, Japan; f Department of Radiology, Kanazawa University Graduate School of Medical Science, Kanazawa, Japan; g Division of Gastroenterology, Hepatology, and Nutrition, The Ohio State University Wexner Medical Center, Columbus, OH; h Department of Laboratory Medicine, Shinshu University, Matsumoto, Japan; i Department of Gastroenterology, Bantane Hospital, Fujita Health University, Nagoya, Japan; j Department of Surgical Pathology, Aichi Medical University Hospital, Nagakute, Japan; k Department of Anatomic Pathology, Kurashiki Central Hospital, Kurashiki, Japan

**Keywords:** AIP, autoimmune pancreatitis, CT, computed tomography, ERC, endoscopic retrograde cholangiography, ERCP, endoscopic retrograde cholangiopancreatography, EUS, endoscopic ultrasound, FNA, fine-needle aspiration, IDUS, intraductal ultrasonography, IgG4, immunoglobulin G4, IgG4-RD, IgG4-related disease, IgG4-SC, IgG4-related sclerosing cholangitis, MRCP, magnetic resonance cholangiopancreatography, MRI, magnetic resonance imaging, PSC, primary sclerosing cholangitis

## Abstract

Biliary strictures caused by inflammation or fibrosis lead to jaundice and cholangitis which often make it difficult to distinguish malignant strictures. In cases when malignancy cannot be excluded, surgery is often performed. The concept of immunoglobulin G4 (IgG4)–related sclerosing cholangitis (SC) as a benign biliary stricture was recently proposed. The high prevalence of the disease in Asian countries has resulted in multiple diagnostic and treatment guidelines; however, there is need to formulate a standardized diagnostic strategy among various countries considering the utility, invasiveness, and cost-effectiveness. We evaluated accuracies of various diagnostic modalities for biliary strictures comparing pathology in the Delphi meetings which were held in Rochester, MN. The diagnostic utility for each modality was graded according to the experts, including gastroenterologists, endoscopists, radiologists, and pathologists from the United States and Japan. Diagnostic utility of 10 modalities, including serum IgG4 level, noninvasive imaging, endoscopic ultrasound, endoscopic retrograde cholangiopancreatography–related diagnostic procedures were advocated and the reasons were specified. Serum IgG4 level, noninvasive imaging, diagnostic endoscopic ultrasound and intraductal ultrasonography under endoscopic retrograde cholangiopancreatography were recognized as useful modalities for the diagnosis. The information in this article will aid in the diagnosis of biliary strictures particularly for distinguishing IgG4–SC from cholangiocarcinoma and/or primary SC.

Inflammatory or fibrotic strictures of the biliary system typically lead to jaundice and cholangitis, which are associated with high patient morbidity. Appropriate treatment is dependent on an accurate clinical diagnosis, which can often be challenging. Surgery is often performed when malignancy cannot be excluded, but this may be avoidable in some circumstances.

The concept of immunoglobulin G4 (IgG4)–related sclerosing cholangitis (SC) as a benign biliary stricture was recently proposed.^1^ IgG4-SC is a distinct type of cholangiopathy characterized by an elevated serum IgG4 level, dense infiltration of IgG4-positive plasma cells and lymphocytes, fibrosis (often storiform in shape), and obliterative phlebitis in the bile-duct wall. IgG4-SC is frequently associated with type 1 autoimmune pancreatitis (AIP) and is recognized as a biliary manifestation of IgG4-related disease (IgG4-RD). The inflammatory component of IgG4-SC improves with induction steroid therapy, and may benefit from maintenance therapy with steroids or other immunosuppressive agents.^2^^,^^3^ Therefore, accurate diagnosis of IgG4-SC is crucial for avoiding unnecessary surgery.

The high prevalence of IgG4-RD in Asian countries has resulted in multiple publications and guidelines; however, there is need to formulate a standardized diagnostic strategy considering the differences in the utility, invasiveness, and cost-effectiveness of diagnostic modalities. Therefore, we convened Delphi meetings of expert gastroenterologists, endoscopists, radiologists, and pathologists from the United States and Japan to discuss diagnostic strategies for biliary strictures, and particularly for distinguishing IgG4-SC from cholangiocarcinoma and/or primary sclerosing cholangitis (PSC). Diagnostic utility for each modality was graded as 1 (high), 2 (medium), or 3 (low) (Table). When recommendations were discordant, we described two grades, and put the more common opinion in front (ie, major opinions were 3 [low] and minor opinions were 2 [medium], we described as “3-2”).

**Table:** Modalities Useful for Diagnosing Biliary Stricture and Distinguishing IgG4-SC From Cholangiocarcinoma or PSC^a^

Modality	Diagnostic utility^b^	Reasons
Blood parameter		
Serum IgG4 level	1	The serum IgG4 level is elevated in 90% of IgG4-SC cases, but levels can also be mildly elevated in other conditions.
Non-invasive imaging		
Contrast-enhanced CT	1	Useful for detecting dilation or wall-thickening biliary abnormalities, metastasis of cholangiocarcinoma, and/or other organ involvement in patients with IgG4-related disease.
MRI/MRCP	1	To detect stenosis or dilation of the bile ducts, an image of the entire biliary system is needed. Cholangiography or pancreatography under MRCP is minimally invasive, but does not always yield diagnostic images.
EUS		
Diagnostic EUS	1	Enables with high accuracy the detection of pancreatic swelling in patients with autoimmune pancreatitis. Furthermore, evaluation of wall thickening with or without stenosis can distinguish IgG4-SC from cholangiocarcinoma.
EUS-FNA of the bile duct	2-3	Not recommended due to the risk of seeding and/or bile leakage, but may be useful if the tumor is large enough to puncture.
ERCP		
Brush cytology from the bile duct	3-2	There is low sensitivity for detecting cholangiocarcinoma, and there is risk for false-positive diagnoses.
Biopsy under ERC from the bile duct	2-3	Biopsy under ERC is useful for diagnosing bile-duct stricture caused by cancer. However, it cannot be used to detect pathological indicators of IgG4-SC^c^ or PSC.
IDUS	1	IDUS findings of strictures and nonstrictures of the bile duct enable differentiation of IgG4-SC from cholangiocarcinoma. Symmetrical wall thickening involves both strictured and nonstrictured portions in IgG4-SC.
Cholangiography	1-2	ERC is useful for differentiating IgG4-SC from PSC. A cholangiogram is required for biliary drainage, and IDUS and biopsy are needed to rule out cholangiocarcinoma.
Cholangioscopy	2	Detailed structures of the bile duct mucosa can be observed. It aids in diagnosis by improving the targeting of bile duct biopsies, but is not diagnostic alone. Further improvement of image quality and establishment of diagnostic criteria based on high-quality images are needed.

a CT, computed tomography; ERC, endoscopic retrograde cholangiography; ERCP, endoscopic retrograde cholangiopancreatography; EUS, endoscopic ultrasonography; EUS-FNA, endoscopic ultrasound-guided fine-needle aspiration; IDUS, intraductal ultrasonography; IgG4-SC, immunoglobulin G4–related sclerosing cholangitis; MRCP, magnetic resonance cholangiopancreatography; MRI, magnetic resonance imaging; PSC, primary sclerosing cholangitis

b Diagnostic utility levels defined as: high, 1; medium, 2; and low, 3.

c We occasionally detect IgG4 plasma cells and inflammatory cell infiltration. However, storiform fibrosis and obliterative phlebitis are rare.

## Lesion Distribution of IgG4-SC

The Japanese clinical practice guidelines for IgG4-SC^1^ classify cholangiograms into the following four types^4^ (Supplemental Figure 1, available online at http://mcpiqojournal.org): type 1 is stenosis only in the lower common bile duct, occasionally misdiagnosed as pancreatic cancer or cholangiocarcinoma; type 2 is stenosis diffusely distributed throughout the intra- and extrahepatic bile ducts, sometimes misdiagnosed as PSC; type 3 is stenosis in both the hilar hepatic region and the lower common bile duct; and type 4 is stricture in only the hilar hepatic region. Types 3 and 4 are occasionally misdiagnosed as cholangiocarcinoma. In a recent Japanese survey, 87% of IgG4-SC cases were associated with AIP.^5^ That is, most patients with IgG4-SC have stenosis in the lower common bile duct.

Stricture of the lower common bile duct can be caused by compression due to AIP. Type 1 IgG4-SC is less common in patients with focal-type AIP with only body and tail involvement compared to head involvement (body and tail, 16% [2 of 12]; head, 93% [41 of 44]; *P*<.001).^6^ However, we believe that type 1 IgG4-SC should be classified as IgG4-SC because: (1) pathological examination of surgical specimens shows IgG4-SC–associated inflammation; (2) some cases have inflammation involving the bile-duct wall, but not the adjacent pancreas; and (3) endoscopic ultrasound (EUS) and intraductal ultrasonography (IDUS) of the bile-duct wall show continuous thickening from the intra- to the extrapancreatic bile duct. It is worthwhile to explicitly clarify that previous studies demonstrating an increased risk for relapse of type 1 AIP in patients with biliary tract involvement differentiate based on distal only (ie, type 1 IgG4-SC) compared to proximal involvement (ie, types 2-4 IgG4-SC), with higher risk in the latter group.

## Serum IgG4 Level

Almost 90% of patients with IgG4-SC have a high serum IgG4 level, which is higher than reported for all patients with type 1 AIP, which is approximately 60%. Although serum IgG4 level is piece of diagnostic evidence for IgG4-SC, its specificity is suboptimal to use in isolation of other findings. IgG4-SC cannot be diagnosed based solely on an elevated serum IgG4 level. From previous reports,^7^^,^^8^ it has been found that the sensitivity and specificity of an elevated serum IgG4 level were 64% to 90% and 87% to 93%, respectively. An elevation of serum IgG4 levels is also seen in 8% to 14% of cholangiocarcinoma patients^7^^,^^8^ and 9% to 22% of PSC patients.^8^, ^9^, ^10^, ^11^, ^12^, ^13^, ^14^, ^15^, ^16^

Based on the above cholangiographic classification, compared to type 1 IgG4-SC, 5.7% of pancreatic cancer patients have a serum IgG4 level higher than the cutoff.^8^ For type 2, 12.7% of PSC patients, and for types 3 and 4, 8.1% of cholangiocarcinoma patients, respectively, have a serum IgG4 level higher than the cutoff.^8^^,^^17^ Therefore, if there is a discrepancy between imaging findings and the serum IgG4 level, we prioritize the former.

The term “diagnostic utility 1” is applied to the serum IgG4 level when it is elevated in 90% of IgG4-SC cases, but levels can also be mildly elevated in other conditions.

## Noninvasive Imaging

The primary role of noninvasive imaging (computed tomography [CT] or magnetic resonance imaging [MRI]/magnetic resonance cholangiopancreatography [MRCP]) for the evaluation of biliary strictures for possible IgG4-SC relates to the ability to identify the involvement of other organs associated with IgG4-RD, particularly classic abnormalities of the pancreatic parenchyma. Although imaging sequelae of cirrhosis may potentially support a diagnosis of PSC, IgG4-SC can also lead to secondary biliary cirrhosis, so this is not specific. The presence of satellite lesions in the liver would clearly favor a diagnosis of cholangiocarcinoma.

Diagnostic utility 1 for contrast-enhanced CT is considered when it is able to detect dilation or wall-thickening biliary abnormalities, metastasis of cholangiocarcinoma, and/or other organ involvement in patients with IgG4-RD.

Diagnostic utility 1 for MRI/MRCP is considered when it is able to detect stenosis or dilation of the bile ducts; an image of the entire biliary system is needed. Cholangiography or pancreatography under MRCP is minimally invasive, but does not always yield diagnostic images.

## Endoscopic Ultrasound

Although commonly performed for the evaluation of patients with known or suspected AIP, the sonographic abnormalities of the pancreas on EUS are not well characterized. Rather, the primary role of EUS is to perform fine-needle aspiration (FNA) to rule out pancreatic malignancy. However, the evaluation of wall thickening with or without biliary stenosis can distinguish IgG4-SC from cholangiocarcinoma. In fact, EUS can often show bile duct wall thickening that is more extensive than visualized with noninvasive imaging. Few reports have described EUS findings of IgG4-SC.^18^, ^19^, ^20^ Du et al^18^ reported that the ability to observe the thickened wall (94.4% [17 of 18 patients]) was significantly more frequent in IgG4-SC cases compared to cholangiocarcinoma. Feng et al^20^ proposed the biliary inflammation scoring method to distinguish IgG4-SC from cholangiocarcinoma. Using the scoring method, the sensitivity, specificity, and accuracy were 86%, 95%, and 90%, respectively. They concluded that the scoring method is a promising diagnostic method to discriminate IgG4-SC. Because of the low risk of complications related to a diagnostic EUS (without FNA), we recommend EUS in cases that are ambiguous despite assessment of the findings of other imaging modalities, such as CT, MRI, and/or MRCP.

EUS-FNA of the bile duct is occasionally useful for diagnosing cholangiocarcinoma, but one must take special care due to the risk of tumor seeding and/or bile leakage. A recent meta-analysis^21^ has revealed that the sensitivity and specificity of EUS-FNA for diagnosis of malignant biliary stricture were 80% and 97%, respectively. But, the role of EUS-FNA for IgG4-SC is unclear. Only one case report^22^ has revealed the usefulness of EUS-FNA for diagnosing IgG4-SC. In the report, the investigators performed EUS-FNA from the common bile duct following the insertion of an endoscopic biliary stent. Limited information was available for the accuracy including adverse events, and further studies are needed to clarify the role of EUS-FNA in diagnosing IgG4-SC.

Diagnostic utility 1 for diagnostic EUS is considered when it enables with high accuracy the detection of pancreatic swelling in patients with AIP. Furthermore, evaluation of wall thickening with or without stenosis can distinguish IgG4-SC from cholangiocarcinoma.

Diagnostic utility 2-3 is considered for EUS-FNA of the bile duct, and it is not recommended due to the risk of seeding and/or bile leakage, but may be useful if the tumor is large enough to puncture.

## Endoscopic Retrograde Cholangiopancreatography

The role of endoscopic retrograde cholangiopancreatography (ERCP) in the diagnosis and management of AIP has evolved over time. In early experience with AIP, most patients underwent ERCP for treatment of jaundice.^23^ However, there are more recent reports demonstrating the ability to successfully treat jaundice with steroid therapy alone in patients with a definitive AIP diagnosis.^24^ Over time, ERCP has come to be used more for the diagnosis of patients with indeterminate features using different accessories, as well as treatment of jaundice in patients with an uncertain diagnosis.

## Brush Cytology

Brush cytology is frequently used to detect bile-duct malignancy, and, in the United States, to rule out malignancy in patients with PSC with a dominant stricture. Although brush cytology enables the detection of malignancy, qualitative diagnosis is difficult, and 3% to 15% of benign cases are reportedly misdiagnosed as cholangiocarcinoma.^25^^,^^26^ Half of the cases that are misdiagnosed by brush cytology are subsequently confirmed to be PSC or IgG4-SC. From previous reports, the sensitivity of brush cytology under endoscopic retrograde cholangiography (ERC) was only 42%, with a negative predictive value of 58%.^27^^,^^28^ Therefore, the role of brush cytology in the evaluation of possible IgG4-SC is limited.

Diagnostic utility 3-2 is considered for brush cytology from the bile duct) as there is low sensitivity for detecting cholangiocarcinoma, and there is risk for false-positive diagnoses.

## Biopsy Under ERC

To diagnose bile-duct stricture caused by cancer, biopsy at the time of ERC can be useful. Although specificity of biopsy under ERC was 97%, sensitivity was only 56%.^28^ It is often not possible to distinguish benign strictures from those caused by neoplasms based on biopsy specimens lacking definitive pathological features of PSC or IgG4-SC due to the small tissue samples. As for diagnosing IgG4-SC by biopsy specimen under ERC, the accuracy rate ranges from 0% to 88% and lacks consistency.^6^^,^^29^, ^30^, ^31^ In patients with IgG4-SC, we occasionally detect IgG4-positive plasma cells and inflammatory cell infiltration, but these are not typical findings. Storiform fibrosis and obliterative phlebitis are rarely present in biopsy specimens, but suggestive of IgG4-SC when present.

Diagnostic utility 2-3 is considered for biopsy under ERC from the bile duct as biopsy under ERC is useful for diagnosing bile-duct stricture caused by cancer. However, it cannot be used to detect pathological indicators of IgG4-SC or PSC.

## Intraductal Ultrasonography

**Figure 1:**
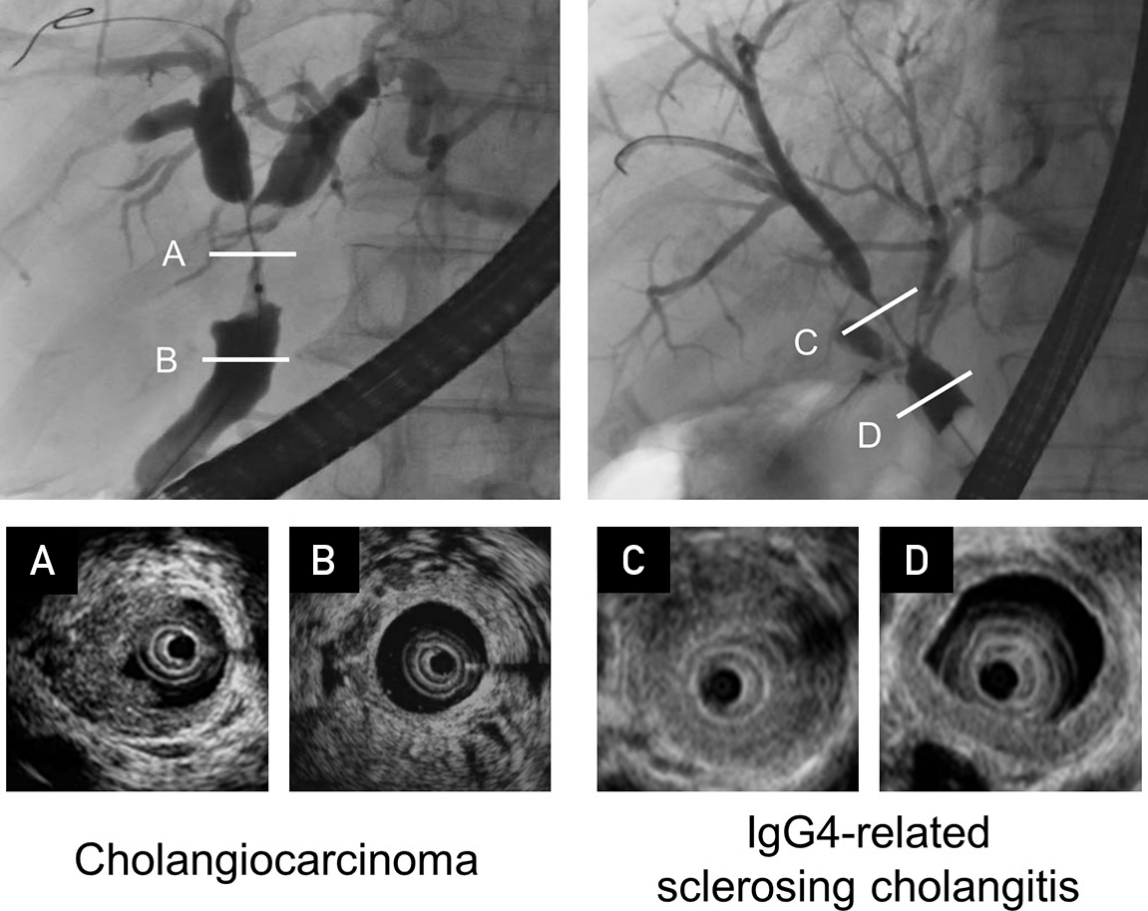
Intraductal ultrasound (IDUS) findings of strictures and nonstrictures of the bile duct enables differentiation of immunoglobulin G4 sclerosing cholangitis (IgG4-SC) from cholangiocarcinoma. There is asymmetrical thickening in strictures associated with cholangiocarcinoma (A), whereas there is symmetrical, concentric thickening observed in IgG4-SC (B). Similarly, there is symmetrical wall thickening in the nonstrictured segments of IgG4-SC (D), which is not present in cholangiocarcinoma (C); this is a specific finding for IgG4-SC (D).

The IDUS findings of strictures and nonstrictured segments of the bile duct can enable the differentiation of IgG4-SC from cholangiocarcinoma (Figure 1). Circular-symmetrical wall thickening spreads from the stricture to the nonstricture portion in patients with IgG4-SC.^19^^,^^31^ Wall thickening in a nonstrictured segment is specific to IgG4-SC.^31^ It is possible to evaluate the bile duct for horizontal sectional view, which reveals symmetric wall thickening more easily and precisely than vertical view. In cases of biliary inflammation or after biliary drainage, modifications of the bile duct may be present.

Diagnostic utility 1 is considered for IDUS because IDUS findings of strictures and non-strictures of the bile duct enable differentiation of IgG4-SC from cholangiocarcinoma. Symmetrical wall thickening involves both strictured and nonstrictured portions in IgG4-SC.

## Cholangiography

ERC is useful for diagnosing IgG4-SC, and particularly for its differentiation from PSC.^32^^,^^33^ The strictures in patients with IgG4-SC are longer than those in PSC patients, and the beaded appearance typical of PSC is rare. MRCP is minimally invasive and has no risk of cholangitis. However, an ERC is required in patients with an unclear diagnosis needing biliary drainage, and IDUS and biopsy can be performed simultaneously to rule out cholangiocarcinoma.

Diagnostic utility 1-2 is considered for cholangiography because ERC is useful for differentiating IgG4-SC from PSC. A cholangiogram is required for biliary drainage, and IDUS and biopsy are needed to rule out cholangiocarcinoma.

## Cholangioscopy

**Figure 2:**
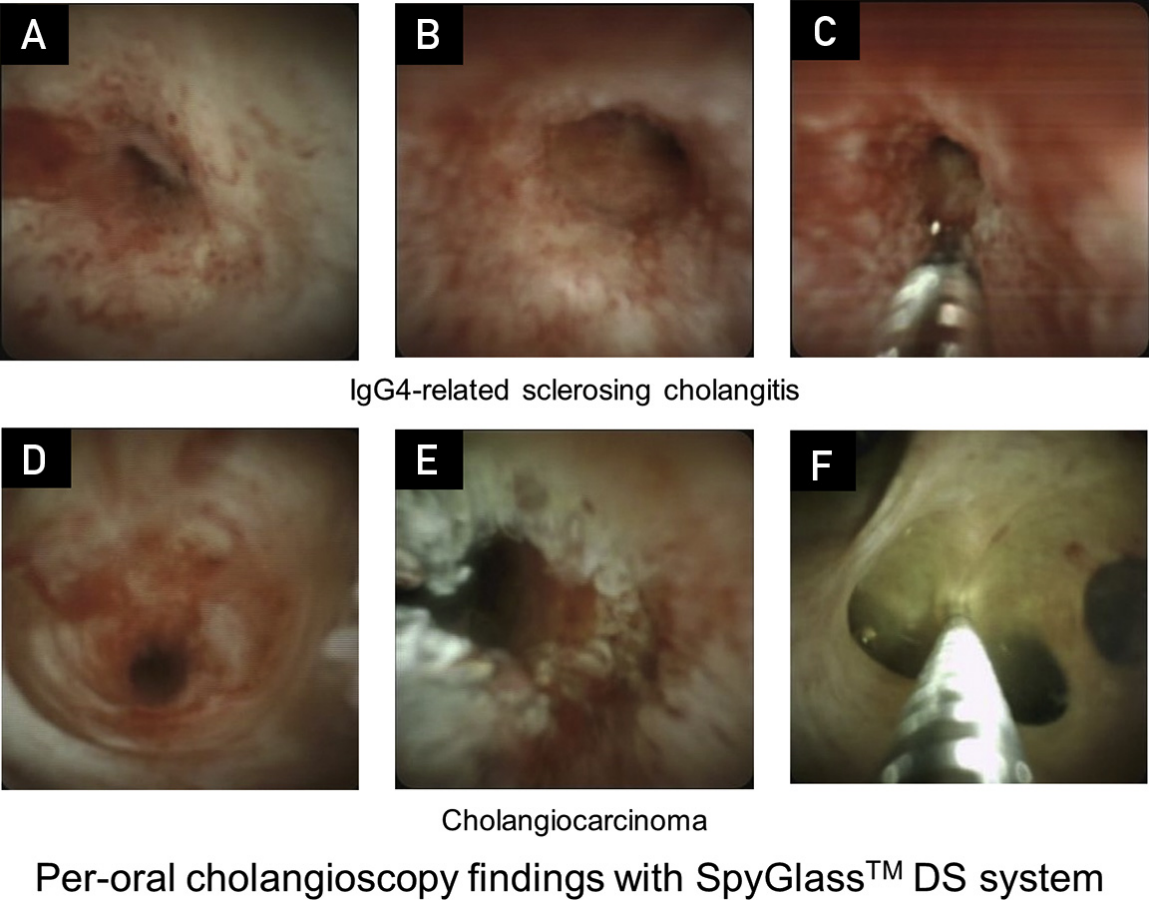
Per-oral cholangioscopy findings with SpyGlass DS system (Boston Scientific Corp, Natick, MA). A,B, Dilated and tortuous vessels could observe in a patient with immunoglobulin G4 sclerosing cholangitis (IgG4-SC). However, in clinical practice, bile duct contact injury caused by guide-wire makes it difficult to diagnose accurately. Partially enlarged vessels (D) and granular change (E) were found in a patient with cholangiocarcinoma. Cholangioscopy is important to improve the targeting of bile duct biopsies and to allow accurate bile duct biopsy from the lesion (C) and/or from normal bile duct for step biopsy (F).

Cholangioscopy enables detailed examination of the structure of the bile-duct mucosa (Figure 2). From previous reports, dilated and tortuous vessels and absence of partially enlarged vessels were typical findings of IgG4-SC.^34^^,^^35^ However, the findings of cholangioscopy alone are not sufficient to make a diagnosis, and are not well established. Diagnosis of lesions by upper^36^ and lower^37^ gastrointestinal magnifying endoscopy is facilitated by the acquisition of detailed information on the surface vascular structure. However, in contrast to magnifying endoscopy, ERC without biopsy is insufficient for diagnosis. Also, in clinical practice, bile duct injury caused by guide-wire make it difficult to diagnose accurately. Further improvement of the image quality and establishment of diagnostic criteria based on high-quality images are needed.

Regardless of the difficulty, cholangioscopy is important to improve the targeting of bile duct biopsies. Therefore, cholangioscopy has a potential to distinguish IgG4-SC from cholangiocarcinoma and PSC.

Diagnostic utility 2 is considered for cholangioscopy as detailed structures of the bile duct mucosa can be observed. It aids in diagnosis by improving the targeting of bile duct biopsies, but is not diagnostic alone. Further improvement of image quality and establishment of diagnostic criteria based on high-quality images are needed.

## Conclusion

Diagnosing biliary strictures is complex but can be facilitated by using complementary modalities. The information in this article will aid in the diagnosis of biliary strictures, particularly IgG4-SC.
