# Supplementary figures and images for: Diagnosing Biliary Strictures: Distinguishing IgG4-Related Sclerosing Cholangitis From Cholangiocarcinoma and Primary Sclerosing Cholangitis
Source: Mayo Clin Proc Innov Qual Outcomes. 2021 Jun 10;5(3):535–41. doi: 10.1016/j.mayocpiqo.2021.03.005 (PMC8240333; doi:10.1016/j.mayocpiqo.2021.03.005)

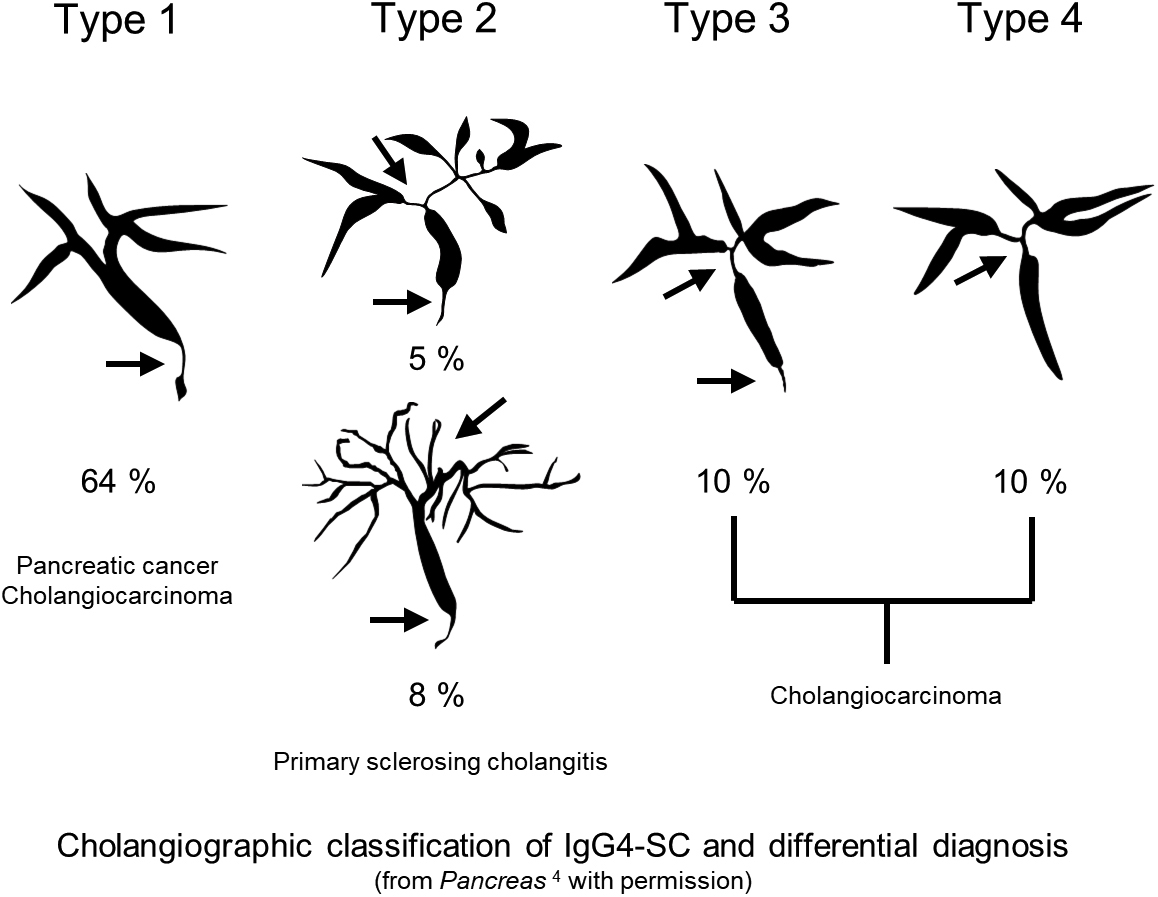

Supplement: Supplemental Figure 1 [file figs1.jpg]
